# From osteoarthritic synovium to synovial-derived cells characterization: synovial macrophages are key effector cells

**DOI:** 10.1186/s13075-016-0983-4

**Published:** 2016-04-04

**Authors:** Cristina Manferdini, Francesca Paolella, Elena Gabusi, Ylenia Silvestri, Laura Gambari, Luca Cattini, Giuseppe Filardo, Sandrine Fleury-Cappellesso, Gina Lisignoli

**Affiliations:** SC Laboratorio di Immunoreumatologia e Rigenerazione Tissutale, Istituto Ortopedico Rizzoli, Via di Barbiano 1/10, Bologna, 40136 Italy; SD Laboratorio RAMSES, Istituto Ortopedico Rizzoli, Bologna, 40136 Italy; Clinica Ortopedica e Traumatologica II, Istituto Ortopedico Rizzoli, Bologna, 40136 Italy; EFS-Pyrénéés-Méditerranéé, Toulouse, F-31300 France

**Keywords:** Osteoarthritis, Synovial fibroblasts, Synovial macrophages, Inflammatory factors, Degradative factors

## Abstract

**Background:**

The aim of the study was to characterize synovial cells from OA synovium with low-grade and moderate-grade synovitis and to define the role of synovial macrophages in cell culture.

**Methods:**

Synovial tissue explants were analyzed for the expression of typical markers of synovial fibroblasts (SF), synovial macrophages (SM) and endothelial cells. Synovial cells at passage 1 (p.1) and 5 (p.5) were analyzed for different phenotypical markers by flow cytometric analysis, inflammatory factors by multiplex immunoassay, anabolic and degradative factors by qRT-PCR. P.1 and p.5 synovial cells as different cell models were co-cultured with adipose stem cells (ASC) to define SM effects.

**Results:**

Synovial tissue showed a higher percentage of CD68 marker in moderate compared with low-grade synovitis. Isolated synovial cells at p.1 were positive to typical markers of SM (CD14, CD16, CD68, CD80 and CD163) and SF (CD55, CD73, CD90, CD105, CD106), whereas p.5 synovial cells were positive only to SF markers and showed a higher percentage of CD55 and CD106. At p.1 synovial cells released a significantly higher amount of all inflammatory (IL6, CXCL8, CCL2, CCL3, CCL5) and some anabolic (IL10) factors than those of p.5. Moreover, p.1 synovial cells also expressed a higher amount of some degradative factors (MMP13, S100A8, S100A9) than p.5 synovial cells. Co-culture experiments showed that the amount of SM in p.1 synovial cells differently induced or down-modulated some of the inflammatory (IL6, CXCL8, CCL2, CCL3, CCL5) and degradative factors (ADAMTS5, MMP13, S100A8, S100A9).

**Conclusions:**

We found that p.1 (mix of SM and SF) and p.5 (only SF) synovial cells represent two cell models that effectively reproduce the low- or moderate-grade synovitis environment. The presence of SM in culture specifically induces the modulation of the different factors analyzed, confirming that SM are key effector cells.

**Electronic supplementary material:**

The online version of this article (doi:10.1186/s13075-016-0983-4) contains supplementary material, which is available to authorized users.

## Background

Osteoarthritis (OA) is defined as a disease of the whole joint because it affects not only the cartilage but the subchondral bone and the synovial tissue that undergo structural and metabolic modifications [1, 2]. Different reports have recognized the importance of synovial inflammation as a key factor associated with the pain and symptoms of OA, even in the early phase of the disease [3–5]. New imaging techniques (ultrasound and magnetic resonance imaging) demonstrate synovitis with effusion in 95 % of patients with OA and synovitis without effusion in 70 % of patients [6]. A recent report has identified a gene expression pattern of cells from inflamed and non-inflamed areas of synovial tissue in OA [7].

Synovial inflammation is a process characterized by synovial thickening (hypertrophy and hyperplasia) and cell infiltration (lymphocytes and macrophages) [8, 9]. Histological analysis of synovium in OA shows an increased number of lining cells and infiltrating cells, mainly consisting of macrophages [8, 10] with a very low percentage of B and T cells [11]. Synovial inflammation is now accepted as an important feature of the symptoms and progression of OA [6].

Normal synovial layers in OA are composed of synovial fibroblasts (SF) and inflammatory leukocytes (lymphocytes and macrophages) [12]. SF are mesenchymal cells that display many characteristics of fibroblasts, including vimentin, CD55, CD90, cadherin-11, vascular adhesion molecule-1 (VCAM-1) and intracellular adhesion molecule-1 (ICAM-1) [13–15]. SF constitutively produce IL6, chemokine (C-X-C motif) ligand (CXCL)8/IL8, chemokine (C-C motif) ligand (CCL)2/monocyte chemotactic protein (MCP-1), transforming growth factor (TGF)β, and fibroblast growth factor [13]. Moreover, synovium was recently reported to contain cells that, after isolation and cell-culture expansion, display a mesenchymal stem cell (MSC) phenotype indistinguishable from SF [15]. Synovial macrophage-like (SM) cells in OA show a phenotype similar to other resident cell macrophages, including CD11b, CD14, CD16 and CD68, and they produce the main inflammatory mediators, such as IL1, IL6, TNFα, matrix metalloproteinases (MMPs) and aggrecanases (ADAMTS), which contribute to articular matrix degradation [16]. Isolated synovial cells in OA are mainly composed of SF with 7 % SM, less than 0.5 % neutrophils and less than 0.1 % T cells [17]. It has been shown that depletion of CD14-positive SM results in a decline in IL1β and TNFα, thus indicating that these cells play a role in inflammation [17]. In the early stage of OA, a unique chemokine signature has been associated with synovial inflammation [3, 18]. CCL5/RANTES and CCL19/macrophage inflammatory protein (MIP)3β chemokines are mainly associated with inflammation [19].

Up to now there has been no in-depth characterization of the synovium and isolated synovial cells in OA. Recent papers [20, 21] have highlighted the importance of better characterization of synovial cells to elucidate the relationship between the different cell types to better define an in vitro cell model. This characterization might lead to better understanding of the interplay between cells in inflammatory and non-inflammatory conditions, to define a synovial cell model as a foundation for devising tailored therapeutic intervention.

To gain new insight into this topic we first analyzed synovial tissue biopsies for the expression of typical markers of SF, SM and endothelial cells and then we followed their expression in isolated synovial cells both at passage 1 (mix of SF and SM), and passage 5 (SF). Subsequently, we performed in-depth analysis of isolated cells for different phenotypical markers, inflammatory, anabolic and degradative factors. Finally, as inflammation induces adipose stem cells (ASC) to exert anti-inflammatory effects [22], we used these cells to test whether in co-culture experiments the presence of SM in synovial cells differently induced or down-modulated some of the inflammatory (IL6, CXCL8/IL8, CCL2/MCP-1, CCL3/MIP1-α, CCL5/RANTES) and degradative factors (ADAMTS5, MMP13, S100A8, S100A9) analyzed. We found that SM in culture induces the specific modulation of the different factors analyzed, thus confirming that SM are key effector cells.

## Methods

### Patient characterization

Synovial tissues were obtained from 26 patients with OA (14 women and 12 men, mean age 66 ± 11.10 years, body mass index 28 ± 4.45 Kg/m^2^, disease duration 7 ± 4.8 years) and Kellgren/Lawrence grade 3/4 [23], who were undergoing total knee replacement surgery. Subcutaneous abdominal fat was obtained from six healthy patients undergoing liposuction. The study was approved by the Rizzoli Orthopaedic Institute ethical committee and all patients provided informed consent (Protocol number 15274).

### Synovial tissue characterization

Synovial tissue specimens were fixed in B5 solution (freshly prepared 9:1 mixture of mercuric-chloride/40 % formaldehyde) at room temperature for 2 h and embedded in paraffin, and serial tissue sections (4 μm thick) from each specimen were prepared and routinely stained with hematoxylin-eosin. The histopathological features of each synovial tissue specimen were evaluated according to the synovitis inflammation scoring system described by Krenn [24], which rank each of the alteration evaluated (hyperplasia of the synovial lining layer, inflammatory infiltrate and stromal cell density) on a scale from 0 to 3. The parameters of synovitis inflammation scoring system were summarized as follows: 0–1 no synovitis; 2–3 low-grade synovitis; 4–6 moderate-grade synovitis; and 7–9 high-grade synovitis. The scoring was performed by two independent observers (CM and GL).

### Immunohistochemical analysis of synovial tissue

Serial sections were incubated overnight at 4 °C with monoclonal anti-human-CD55 (2.5 μg/ml, Millipore, Temecula, CA, USA), −CD68 (10 μg/ml, Dako Cytomation, Denmark), −Factor VIII (10 μg/ml Dako), −CCL3/MIP1α (2.5 μg/ml R&D Systems, Minneapolis, MN, USA) and -S100A8 (4.5 μg/ml R&D) diluted in Tris-buffered saline (TBS) containing 0.1 % bovine serum albumin (BSA). Samples were then rinsed in TBS and sequentially incubated at room temperature for 20 minutes with multilinker biotinylated secondary antibody (Biocare Medical, Walnut Creek, CA, USA) and alkaline phosphatase-conjugated streptavidin (Biocare Medical). The reactions were developed using fast red substrate (Biocare Medical), counterstained with hematoxylin, and mounted in glycerol gel. Negative controls were performed using isotype control (Dako Cytomation). Semiquantitative analysis of immunohistochemically stained slides were performed on 20 microscopic fields (×200 magnification) for each section. The analysis was performed using Red/Green/Blue (RGB) with Software NIS-Elements and Eclipse 90i microscope (Nikon Instruments Europe BV). Briefly, we acquired the total number of blue-stained nuclei and the total number of positive-stained red cells. The data were expressed as percentage of positive cells for CD55 and CD68, respectively. For Factor VIII analysis we counted the number of positive vessels in 20 microscopic fields. The data were expressed as the mean number of positive vessels/5 mm^2^ area.

### Isolation and characterization of synovial cells from non-digested fragments

Synovial cells were isolated following a standardized procedure as previously described^.^ [25] and were used for the experiments at both passages 1 and 5. Briefly, synovial tissue was fragmented and the pieces cultured for 7 days. Cultured synovial fragments were removed and fixed as described above. Synovial cells were grown in OPTIMEM culture medium (Life Technologies Italia, Monza, Italy) supplemented with 100 U/ml penicillin and 100 μg/ml streptomycin in a humidified atmosphere, at 37 °C with 5 % CO_2_.

Synovial cells at both passages 1 (p.1) and 5 (p.5) were characterized by flow cytometry using the following markers expressed by SF (CD55 (2.5 μg/ml, Millipore), CD73, CD90, and CD105 (5 μg/ml, BD Pharmingen, San Jose, CA, USA), CD106 (10 μg/ml, Millipore), SM (CD14 and CD16 (5 μg/ml, Dako), CD68 (5 μg/ml, BD), CD80 (2 μg/ml, GeneTex Inc., Irvine, CA, USA), and CD163 (10 μg/ml, Abcam, Cambridge, UK)), and by endothelial (CD31, 2 μg/ml, R&D) and mononuclear cells (CD3, CD34, and CD45 (5 μg/ml, Dako). Briefly, after harvesting cells upon detachment, they were washed twice with PBS, centrifuged, and washed in a flow cytometry buffer (PBS supplemented with 2 % BSA and 0.1 % sodium azide).

Aliquots of 1 × 10^5^ cells were then incubated with primary antibodies at 4 °C for 30 minutes, washed twice with a flow cytometry buffer, and incubated with polyclonal rabbit anti-mouse immunoglobulins/fluorescein isothiocyanate (FITC) conjugate (Dako Cytomation) at 4 °C for 30 minutes. After two final washes, the cells were analyzed using a fluorescence-activated cell sorting (FACS) CantoII Cytometer (Becton Dickinson). For isotype control, non-specific mouse IgG was substituted for the primary antibody.

### Passage 1 and 5 synovial cells as specific cell models for cell co-culture

Synovial cells at both passages 1 and 5 (100,000 cells/well) were seeded in the lower chamber of a 6-well plate and co-cultured with clinical grade ASC (100,000 cells in Transwells®) for 7 days (medium was changed at day 2) in complete DMEM using a defined cell ratio (1:1) that assures no cell proliferation, as we previously reported [22]. ASC were isolated from subcutaneous abdominal fat according to Good Manufacturing Practice (GMP) [26], and were grown in αMEM supplemented with platelet lysate (PLP) and characterized for the CD markers CD14, CD34, CD45, CD73, CD90 (5 μg/ml, BD Pharmingen) and CD13 (1 μg/ml, eBioscience, San Diego, CA, USA) as we previously described [22, 26] (data not shown). Control cells were mono-cultures of ASC and synovial cells at both passages 1 and 5. The cells were harvested on day 7 for quantitative RT-PCR analysis and supernatant stored at −80 °C. The concentrations of IL6, CXCL8/IL8, CCL2/MCP-1, CCL3/MIP1-α, and CCL5/RANTES were analyzed in the supernatant for all conditions tested as described above.

### Cytokine and chemokine release in supernatants

The concentrations of IL1β, IL4, IL6, CXCL8/IL8, IL10, CCL2/MCP-1, CCL3/MIP1α, CCL5/RANTES, TGFβ, and TNFα were simultaneously evaluated in the supernatants of ASC, synovial cells at both passages (1 and 5) in mono- and co-cultures, using multiplex bead-based sandwich immunoassay kits (BioRad Laboratories Inc., Segrate, Italy) following the manufacturer’s instructions. Briefly, we added 50 μl to each well of the diluted standards (fourfold dilution series), controls, and samples in triplicate and added 50 μl of coupled beads, and the plate was incubated at room temperature for 30 minutes. The plate was then washed three times with 100 μl of wash buffer and incubated with 25 μl of detection antibodies for 30 minutes. Finally, the plate was washed three times and incubated with 50 μl of streptavidin-PE for 30 minutes and measured in a reader (Luminex Bio-plex system, Bio-Rad Laboratories Inc.).

### Real-time quantitative reverse transcription polymerase chain reaction (qRT-PCR) analysis

Total RNA was extracted from human ASC, synovial cells in mono- and co-cultures, using RNA PURE reagent (Euroclone Spa, Pero, Italy) according to the manufacturer’s instructions, and then was treated with DNase I (DNA-free Kit, Life Technologies). Reverse transcription was performed using SuperScript VILO (Life Technology) reverse transcriptase and random hexamers, following the manufacturer’s protocol.

Forward and reverse oligonucleotides for PCR amplification of ADAMTS4, ADAMTS5, MMP13, S100A8, and S100A9 are described in Table 1, and real-time PCR was run as previously described [22]. All primer efficiencies were confirmed to be high (>90 %) and comparable (Table 1). For each target gene, messenger RNA (mRNA) levels were calculated, normalized to RPS9 according to the formula 2^-∆Ct^, and expressed as a percentage of the reference gene, as this was expressed in the same amount in all conditions tested.

**Table 1:** Oligonucleotide primers used for real-time polymerase chain reaction

Target gene	Primers (forward and reverse)	Product size (bp)	GenBank accession number	Primer efficiency (%)
*RPS9*	GATTACATCCTGGGCCTGAA ATGAAGGACGGGATGTTCAC	161	NM_001013	94.5
*ADAMTS4*	CTGCCTACAACCACCG GCAACCAGAACCGTCC	293	NM_005099.4	99.1
*ADAMTS5*	GCACTTCAGCCACCATCAC AGGCGAGCACAGACATCC	187	NM_007038.3	92.4
*MMP13*	TCACGATGGCATTGCT GCCGGTGTAGGTGTAGA	277	NM_002427	94.5
*S100A8*	TAGAGACCGAGTGTCCTCA CGCCCATCTTTATCACCAGA	126	NM_002964.4	93.4
*S100A9*	CCATCATCAACACCTTCCACCA CTGCTTGTCTGCATTTGTGTCC	179	NM_002965.3	91.4

### Statistical analysis

Statistical analysis was performed using non-parametric tests because the data did not have a normal distribution (Kolmogorov-Smirnov test). Friedman’s analysis and Dunn’s post hoc test was used to analyze more than two groups of paired data, the Mann–Whitney *U* test was used to analyze unpaired two-group data and the Wilcoxon test was used to analyze paired two-group data. Groups with small samples were evaluated using the exact method. Values were expressed as the median and interquartile range. CSS Statistica Statistical Software (Statsoft Inc., Tulsa, OK, USA) was used for analysis and values of *p* < 0.05 were considered significant.

## Results

### OA synovial tissue explant characterization

**Fig. 1:**
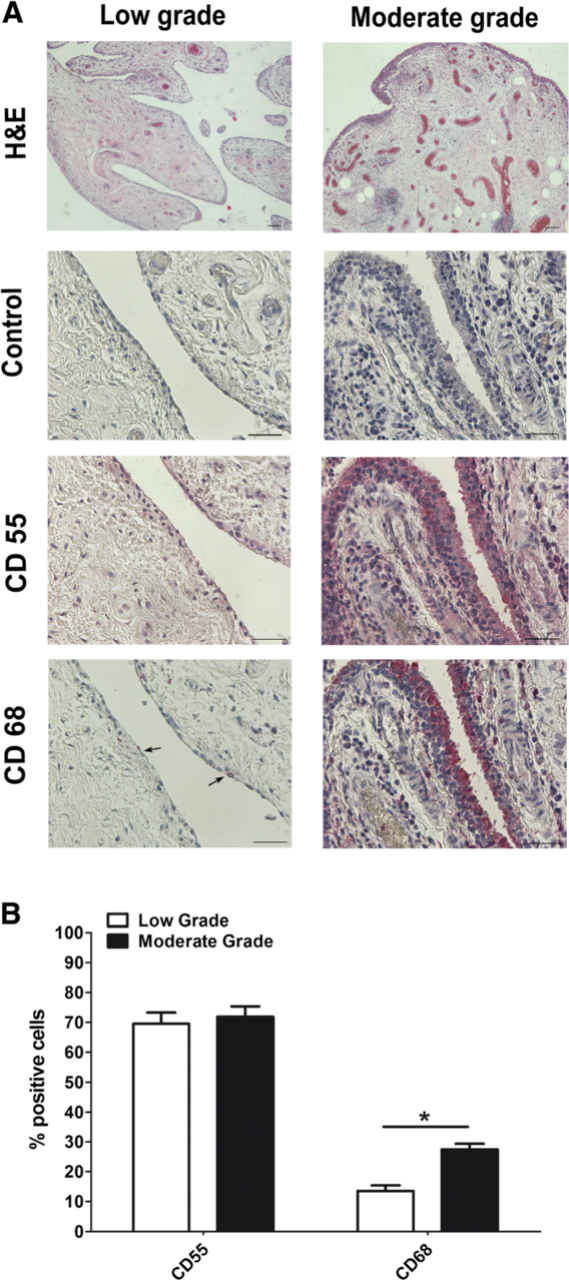
Characterization of synovial tissue in osteoarthritis (OA). **a** Representative samples with low-grade (*left*) and moderate-grade (*right*) OA synovitis, stained with hematoxylin-eosin (*H&E*). *Bars* 100 μm (magnification × 40). Immunohistochemical analysis of CD55 and CD68 on representative cases with low-grade (*left*) and moderate-grade (*right*) synovitis in OA. Negative control for CD55 and CD68 (*Control*). *Bars* 50 μm. **b** Percentage of positive cells to CD55 and CD68 analyzed in both low-grade (n = 4) and moderate-grade (n = 22) synovitis in OA. Data are expressed as the median and interquartile range. *Significant differences between low-grade and moderate-grade synovitis: *p* < 0.005

Synovial tissue explants from 26 patients with OA were first scored on hematoxylin-eosin-stained slides, as reported by Krenn [24] and we found low-grade synovitis in 4 samples and moderate-grade synovitis in 22 samples. Vessel proliferation was also evaluated on Factor VIII-stained slides and there were fewer positive vessels (90 ± 35/5 mm^2^ area) in low-grade than in moderate-grade synovitis (222 ± 79/5 mm^2^ area). Then, for in-depth analysis of the main synovial cell populations present in the synovial tissue, we analyzed CD68 and CD55 to establish the percentage of synovial macrophages and synovial fibroblasts, in both low- and moderate-grade synovitis. As shown in Fig. 1a, CD68 was mainly positive on synovial macrophages located in the lining layer and on a few in the sublining layer (Additional file 1). There were approximately 13 % and 27 % of CD68-positive cells in low- and moderate-grade synovitis, respectively (Fig. 1b). The CD55 typical marker of synovial fibroblast was positive both on the sublining and lining layers (Fig. 1a) (Additional file 1) and was approximately 70 % positive in both low- and moderate-grade synovitis (Fig. 1b).

### Synovial cells characterization

**Fig. 2:**
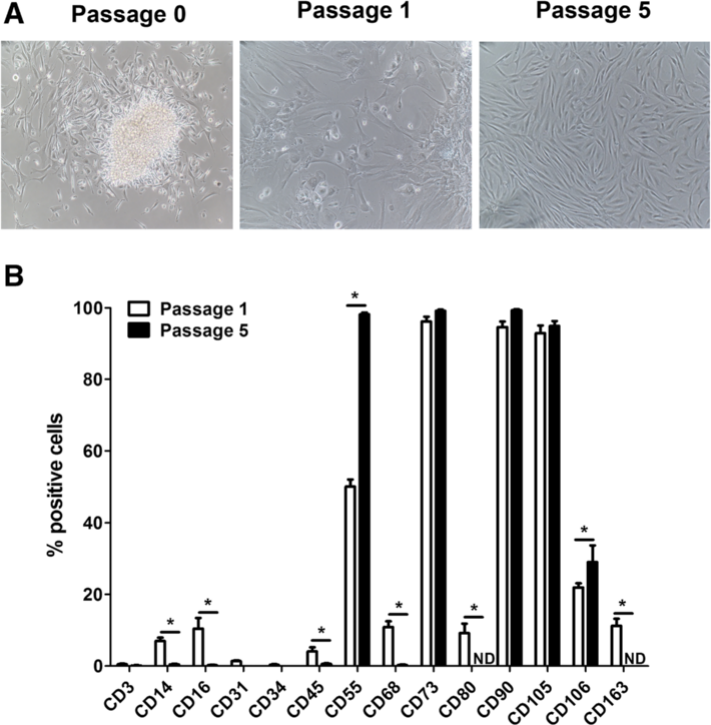
Evaluation of isolated passage 1 and passage 5 synovial cells from moderate-grade synovitis in osteoarthritis. **a** Outgrowth of synovial cells from synovial non-digested fragments (*Passage 0*). Passage 1 synovial cells characterized by a mix of cells with a spindle and a polygonal-star shape. Passage 5 synovial cells characterized only by spindle-shaped morphology. **b** CD3,CD14, CD16, CD31, CD34, CD45, CD55, CD68, CD73, CD80, CD90, CD105, CD106, and CD163 immunocytochemical staining on passage 1 and passage 5 synovial cells analyzed by flow cytometry. Data are expressed as the median and interquartile range (n = 22). *Significant differences between passage 1 and passage 5 synovial cells: *p* < 0.005. *ND* not detected

The cells outgrowing from cultured-synovium tissue fragments (Fig. 2a) were then morphologically and phenotypically analyzed. As shown in Fig. 2a, at p.0 cells started outgrowth from synovial tissue fragments; at p.1 we found two cell types, one spindle-shaped (defined as synovial fibroblasts, SF) and one with polygonal-star morphology (defined as synovial macrophages, SM), whereas at p.5 all cells had only spindle-shaped morphology. Moreover, to confirm that SM and SF had these peculiar cell morphologies, we immunocytochemically stained the isolated p.1 and p.5 cells with anti-CD68 and anti-CD55, typical markers of SM and SF, respectively (data not shown).

These cells at both passages (p.1 and p.5), were then characterized by flow cytometry for markers expressed by SF (CD55, CD73, CD90, CD105, and CD106), SM (CD14, CD16, CD68, CD80, and CD163), endothelial cells (CD31), and mononuclear cells (CD3, CD34, and CD45). As shown in Fig. 2b, p.1 synovial cells had a very low percentage (<3 %) of CD3, CD31, CD34, and CD45, an intermediate percentage (10–20 %) of CD14, CD16, CD68, CD80, CD106 and CD163, and a high percentage (60–100 %) of CD55, CD73, CD90, and CD105. Interestingly, CD80 and CD163 were expressed (approximately 12 %) only by p.1 synovial cells. Conversely, p.5 synovial cells had a very low or negative percentage of all the markers analyzed except for CD55, CD73, CD90, CD105 and CD106. In particular, CD55 and CD106 were the only markers more highly expressed by p.5 synovial cells.

### Factors released by OA synovial cells

**Fig. 3:**
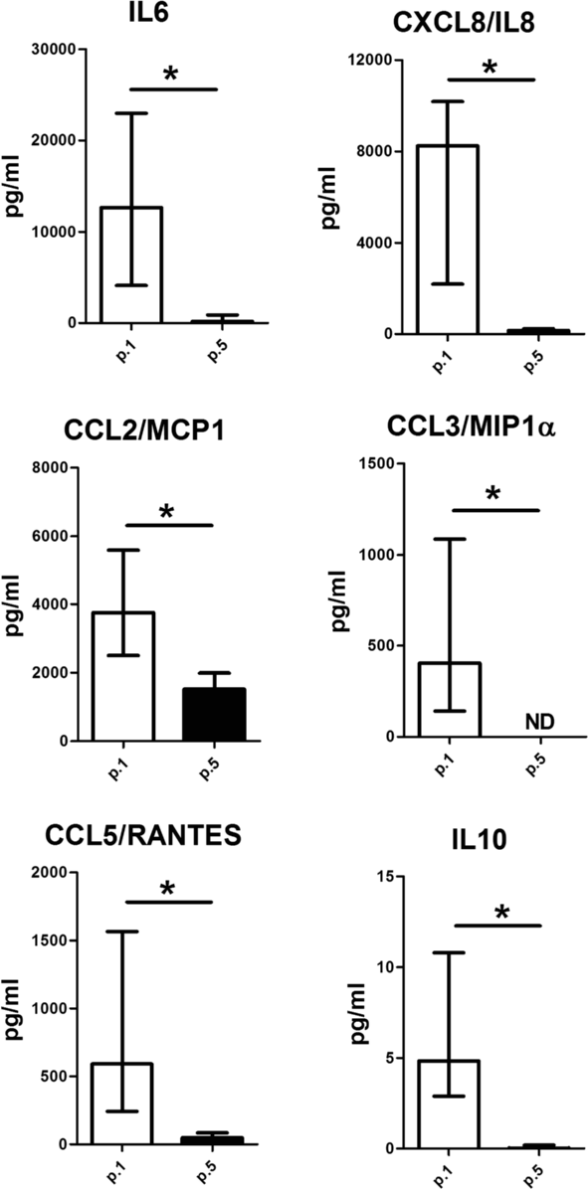
Evaluation of inflammatory and anabolic factors released by passage 1 (*p.1*) and passage 5 (*p.5*) synovial cells from osteoarthritic moderate-grade synovitis. IL6, CXCL8/IL8, CCL2/monocyte chemotactic protein 1 (*MCP-1*), CCL3/macrophage inflammatory protein 1α (*MIP1α*), CCL5/RANTES, and IL10 were evaluated in the supernatant of both p.1 and p.5 synovial cells as described in “Methods”. Data are expressed as the median and interquartile range (n = 22). *Significant differences between synoviocytes at p.1 and p.5: *p* < 0.005

We subsequently evaluated inflammatory factors (IL1β, TNFα, IL6, CXCL8/IL8, CCL2/MCP-1, CCL3/MIP1α, and CCL5/RANTES) and anabolic factors (TGFβ, IL4, and IL10) released by p.1 and p.5 OA synovial cells. As shown in Fig. 3, p.1 synovial cells produced significantly more IL6, CXCL8/IL8, CCL2/MCP-1, CCL3/MIP1α, CCL5/RANTES, and IL10 than those at p.5. IL1β, TNFα, TGFβ and IL4 were not detected at either passage (p.1 or p.5). In particular, p.1 synovial cells released more IL6, CXCL8/IL8, and CCL2/MCP-1 than CCL3/MIP1α, CCL5/RANTES, and IL10. Interestingly, CCL2/MCP-1 was the most abundant factor released by p.5 synovial cells, whereas there was less IL6, CXCL8/IL8, and CCL5/RANTES. IL10 and CCL3/MIP1α from p.5 synovial cells were at the limit of detection or not detected, respectively.

### OA synovial cell degradative factors

**Fig. 4:**
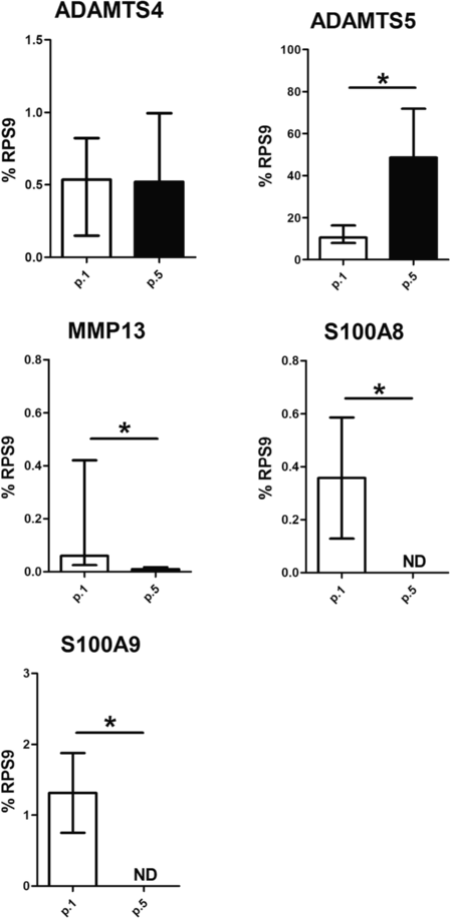
Evaluation of degradative factors expressed by passage 1 (*p.1*) and passage 5 (*p.5*) synovial cells from osteoarthritic moderate-grade synovitis. ADAMTS4, ADAMTS5, MMP13, S100A8, and S100A9 were evaluated in the supernatant of both p.1 and p.5 synovial cells as described in “Methods”. Data are expressed as the median and interquartile range (n = 22). *Significant differences between p.1 and p.5 synovial cells: *p* < 0.005. *ND* not detected

Then we analyzed different factors (ADAMTS4, ADAMTS5, MMP13, S100A8, S100A9) involved in the degradation of joint tissue. As shown in Fig. 4, both synovial cells at p.1 and p.5 expressed the same level of ADAMTS4, whereas there was significantly more ADAMTS5 expressed in p.5 than in p.1 synovial cells. Conversely, MMP13, S100A8, and S100A9 were highly expressed in p.1 synovial cells, but there was very low expression of in MMP13 in p.5 synovial cells, and S100A8 and S100A9 were not detected.

### Synovial macrophages influence cell co-culture effects

**Fig. 5:**
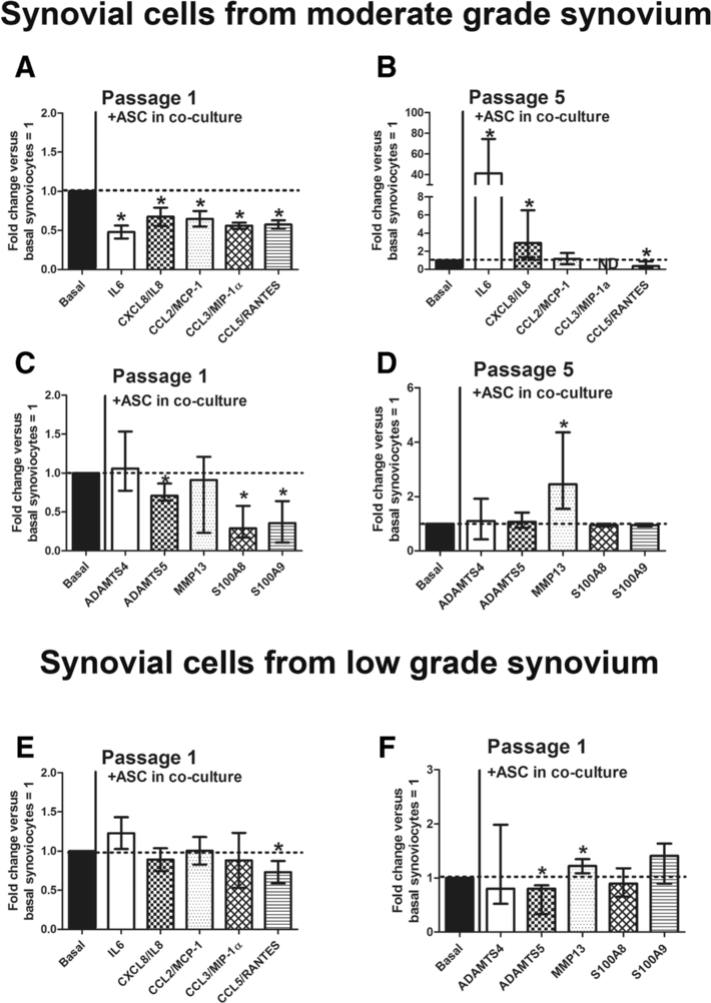
Evaluation of the role of macrophage in cell co-cultures. **a**-**d** Co-culture of adipose stem cells (*ASC*) with passage 1 (p.1) (**a**, **c**) and passage 5 (p.5) (**b**, **d**) synovial cells from osteoarthritic moderate-grade synovitis and evaluation of released inflammatory (IL6, CXCL8/IL8, CCL2/MCP-1, CCL3/MIP1α, and CCL5/RANTES) and expressed degradative factors (ADAMTS4, ADAMTS5, MMP13, S100A8, and S100A9). Data are represented as fold changes versus basal synoviocytes = 1 and expressed as the median and interquartile range (n = 22). *Significant differences between p.1 and p.5 synovial cells: *p* < 0.005. **e**-**f** Co-culture of ASC with p.1 synovial cells from low-grade osteoarthritic synovitis and evaluation of released inflammatory (IL6, CXCL8/IL8, CCL2/MCP-1, CCL3/MIP1α, and CCL5/RANTES) (**e**) and expressed degradative factors (ADAMTS4, ADAMTS5, MMP13, S100A8, and S100A9) (**f**). Data are represented as fold changes versus basal synoviocytes = 1 and expressed as the median and interquartile range (n = 4). *Significant differences between p.1 and p.5 synovial cells: *p* < 0.005

The presence of SM in p.1 synovial cells significantly increased the release of inflammatory, anabolic and degradative factors, thus creating a significantly different milieu from p.5 synovial cells. Therefore, as p.1 and p.5 synovial cells represent two different cell culture models, we tested whether they could differently affect another cell type in co-culture. We chose adipose stem cells (ASC) as the cell model because they are reportedly activated by an inflammatory environment [22]. We analyzed inflammatory and degradative factors, previously tested in basal conditions in p.1 and p.5 synovial cells (Figs. 3 and 4), after co-culture with ASC. We did not test IL10 because ASC express and release large amounts of this cytokine, but did not express the other factors analyzed (data not shown). As we previously reported [22], we confirmed that ASC in co-culture with p.1 synovial cells reduced the release of IL6, CXCL8/IL8, CCL2/MCP-1, CCL3/MIP1α, and CCL5/RANTES (Fig. 5a). Conversely, as shown in Fig. 5b, the co-culture of ASC with p.5 synovial cells (only SF) differently affected the release of the factors evaluated, thus indicating direct dependence by the presence of SM in culture. In particular, ASC when co-cultured with p.5 synovial cells were able to increase the release of IL6 and CXCL8/IL8, however they were unable to affect or significantly decreased, the release of macrophage-like chemokines, such as CCL2/MCP-1 and CCL5/RANTES, respectively. Interestingly, as shown in Fig. 5b, on p.5 synovial cells the ASC were unable to modulate CCL3/MIP1α that was still not released or expressed (data not shown) by SF. Moreover, as shown in Fig. 5c the analysis of degradative factors revealed that ASC on p.1 synovial cells decreased the expression of ADAMTS5, S100A8, and S100A9, but ADAMTS4 and MMP13 were not affected. Conversely, ASC on p.5 synovial cells induced the expression of MMP13 and did not modulate the other factors analyzed (Fig. 5d).

Moreover, to confirm that the effects observed were directly dependent on the amount of SM in synovial cells p.1 we also evaluated all the inflammatory and degradative factors in p.1 synovial cells isolated from low-grade synovial explants, which, as shown in Fig. 1b, contain a very low percentage of SM (CD68 positive). As shown in Fig. 5e-f, ASC were unable to reduce the inflammatory and degradative factors in p.1 synovial cells from low-grade synovitis, except for CCL5/RANTES and ADAMTS5, but MMP13 was induced.

### Immunohistochemical analysis of CD68, CCL3/MIP1α and S100A8 on synovial tissue

**Fig. 6:**
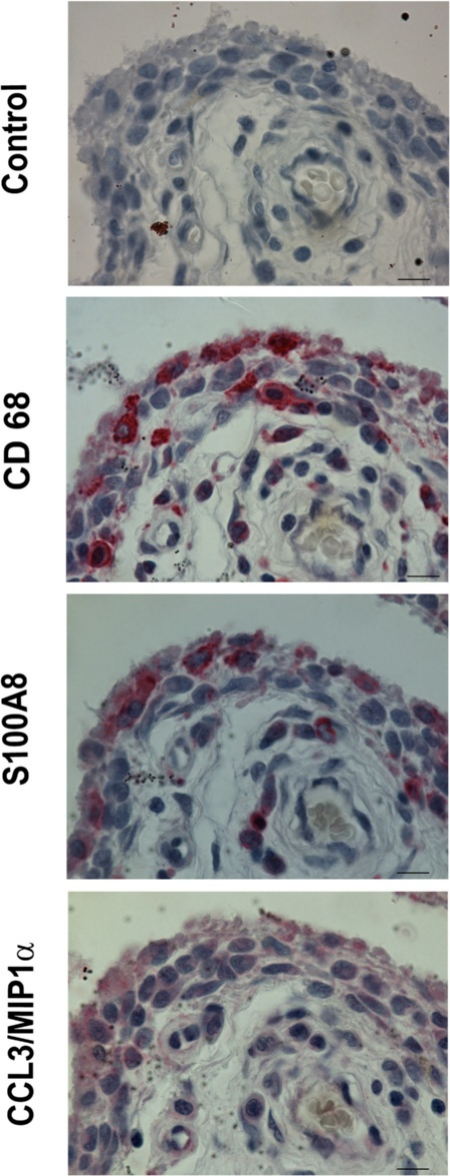
Synovial tissue analysis for macrophage markers. Immunohistochemical analysis of CD68, CCL3/MIP1α, and S100A8 on serial sections for one representative case with moderate-grade osteoarthritic synovitis. *Bars* 10 μm

Furthermore, to confirm that CCL3/MIP1α and S100A8 were specific markers of SM, we also immunostained serial sections of synovial tissue from patients with moderate synovitis using the positive control macrophage marker CD68. As shown in Fig. 6, we confirmed that SM positive to CD68 were also positive to CCL3/MIP1α and S100A8, which we also detected only on p.1 synovial cells. Positive cells were mainly located on the lining layer and around the vessels.

## Discussion

Synovitis is a typical feature in a high percentage of patients with OA, even in the early phase of the disease [3]. Hyperplasia in the synovium is associated with an increased number of synovial lining cells in OA, accompanied by infiltration of inflammatory cells mainly consisting of macrophages [8]. Synovial tissue is a complex structure mainly composed of SF and SM, and in vitro cell models have mainly focused on SF. Therefore, we characterized synovial tissue from low-grade and moderate-grade synovitis and synovial cell outgrowth from cultured non-digested synovial fragments. These cells were analyzed at two cell passages (p.1, a mix of SF and SM and p.5, only SF) to define their phenotype, inflammatory and degradative factors and their functional role.

Our data show that synovium from patients with OA and moderate synovitis had approximately 27 % CD68 (SM) and 70 % CD55 (SF) positive cells, which are well-standardized markers for SM and SF, respectively. The synovial cells outgrowing in culture were evaluated at both p.1 and p.5. SF (p.5) were not positive to typical endothelial (CD31), hematopoietic (CD3, CD34, and CD45) and macrophage-like markers (CD14, CD16, CD68, CD80, and CD163) but they expressed more CD55 and CD106, which are considered relative specific markers of SF [13]. Moreover, SF at both p.1 and p.5 also expressed increased amounts of CD73, CD90 and CD105, which are considered typical markers for identifying mesenchymal stem cells (MSC). In line with several reports [14], the isolation of MSC and SF from the synovium is mainly based on the adhesion properties of the mononuclear cell fraction in vitro, from which they can be cultured-expanded as SF. However, detailed studies, as recently underlined by De Bari [15], are necessary to determine whether synovium-MSC are SF or different subsets, as in culture they are indistinguishable and no markers permit their selective identification. Only p.1 synovial cells were positive to typical macrophage markers, such as CD14, CD16, and CD68 and to some specific M1 or M2 macrophage markers, such as CD80 and CD163, thus showing the contemporary presence of pro-inflammatory M1 type and anti-inflammatory/regenerative M2 type in the OA synovium. Moreover, in contrast to other reports, we found the presence of macrophages in p.1 synovial cell culture, which has been reported to be reduced or absent in synovium-derived cells from digested fragments, and this confirms that enzymatic digestion affects the recovery of the different cell populations present in the synovium [14, 20]. Interestingly, p.1 synovial cells express only very small amounts of CD31, a typical endothelial marker, thus suggesting that this in vitro procedure does not ensure their isolation.

We found that p.1 synovial cells (mix of SF and SM) release significantly more IL6, CXCL8/IL8, CCL2/MCP-1, CCL3/MIP1α, CCL5/RANTES, and IL10 than those of p.5 (SF), thus suggesting that the absence of SM significantly reduces inflammation and does not induce the anabolic factor IL10. It has been shown that CCL2/MCP1 enhances CD106 [27]; our data show an increased number of positive CD106 on p.5 synovial cells that might be induced by CCL2/MCP1. In fact, even if CCL2/MCP1 is reduced compared to that of p.1 synovial cells, it is released in a greater quantity by p.5 synovial cells.

Bondeson et al. [17] reported that depletion of CD14-positive SM was associated with a decline in inflammation associated with decreased IL6, CXCL8/IL8, and CCL2/MCP-1, which is in line with our data in vitro. Among the factors analyzed, we found that S100A8, S100A9, and CCL3/MIP1α were the only undetectable factors (both at molecular and protein level) in p.5 synovial cells, a cell culture characterized by the absence of SM, thus confirming that they are specific markers of SM.

Synovial cells p.1 and p.5 represent two different in vitro cell models characterized by the presence and absence of SM, respectively, which are directly responsible for the low- and high-level inflammatory/degradative milieu. Different studies [28, 29] have used SF (p.5 synovial cells) as a cell model to test drugs or the effects of other cell types, without taking into consideration that synovial tissue is mainly composed of at least two cell types, SM and SF. It is has been known that OA SM are those mainly responsible for synovial inflammation [5, 16, 30] and it has been reported that inflammation induces ASC to exert an anti-inflammatory effect [22, 31, 32]. Therefore, to better define how the presence or absence of SM in cell culture influences the effects observed we evaluated the effects of ASC on inflammation and degradative factors co-cultured with the synovial p.1 and p.5 cell models. We found that in contrast with the anti-inflammatory effects found on p.1 synovial cells from moderate-grade OA, on p.5 synovial cells the ASC modulated the analyzed inflammatory factors differently. In particular, ASC significantly induced IL6 and CXCL8/IL8, decreased CCL5/RANTES, and did not modulate CCL2/MCP-1 or CCL3/MIP1α, which were still not expressed or released, thus demonstrating and confirming a specific dependence of this chemokine on SM and not on SF. Moreover, it is interesting to note that among the inflammatory factors analyzed, the CCL5/RANTES chemokine, mainly associated with a signature of synovial inflammation, was the only one that was still down-modulated in the presence of ASC, both in co-culture with p.1 with low- or moderate-grade synovitis, and with p.5 synovial cells, thus highlighting that this was an effect independent of the presence of SM.

It has been shown that CCL3/MIP1α has an important role in the recruitment of infiltrating leukocytes in the arthritic joint. Moreover, the CCL3-null mouse arthritis model is associated with a reduction of infiltrating cells and normal appearance of the synovium and cartilage, and the absence of pannus or bone resorption, thus confirming the important role of this chemokine in OA [33]. This evidence is also corroborated by a report of increased expression and secretion of CCL3-MIP1α when SF were co-cultured with activated leukocytes (monocytes or polymorphonuclear neutrophils) [34].

Our data show that ASC in co-culture with p.1 synoviocytes from moderate-grade OA decreased the typical inducible factors ADAMTS5, S100A8, and S100A9, and did not affect ADAMTS4 and MMP13. Conversely, in co-culture with p.5 synovial cells they only induced the expression of MMP13, thus suggesting that in the absence of SM, SF appear to acquire a characteristic more typical of SM, such as increased expression of MMP13. Interestingly, we also found that S100A8 and S100A9, the main catabolic factors produced by activated macrophages, were not detected in p.5 synovial cells co-cultured with ASC, thus confirming their specific expression on SM. These data were also confirmed on moderate-grade OA synovial tissue, where we found that S100A8 and CCL3/MIP1α were co-expressed with CD68, the typical macrophage marker.

Moreover, we found that S100A8 and S100A9 inhibition in p.1 synovial cells was also associated with IL6 and CXCL8/IL8 inhibition, which, as already reported [35], are strictly dependent. These data are also in line with a recent report that in a murine collagenase-induced OA model, ASC inhibited synovial activation mainly by reducing S100A8 and S100A9 [36]. Furthermore, large quantities of these catabolic factors have also been found in the synovial tissue of patients with OA, and they predict the development of cartilage destruction [37]. Interestingly, we also confirmed that in contrast to p.1 synovial cells obtained from patients with moderate-grade synovitis, p.1 synovial cells from patients with low-grade synovitis are unable to guide the anti-inflammatory and anti-catabolic effects of ASC, as found for p.5 synovial cells, as SM were present in very small numbers.

## Conclusions

In summary, our data from in vitro analysis show the importance of using the correct in vitro cell models to recreate a milieu that closely resembles OA synovial tissue as the target tissue organ. The availability of in vitro cell models (p.1 and p.5 synovial cells) with large or small numbers of SM effectively reflects the different degrees of OA, which are characterized by different degrees of synovial inflammation, giving the opportunity of testing cells, anti-inflammatory drugs, or factors in a well-defined milieu.

## Additional file

Additional file 1: Immunohistochemical analysis of CD55 and CD68 on representative cases with low grade (left) and moderate grade (right) OA synovitis. Bars 10 μm. (TIF 2835 kb)

## Abbreviations

ADAMTS4: ADAM metallopeptidase with thrombospondin type 1 motif, 4
ADAMTS5: ADAM metallopeptidase with thrombospondin type 1 motif, 5
ASC: adipose stem cells
BSA: bovine serum albumin
CCL2/MCP1: chemokine (C-C motif) ligand 2/monocyte chemotactic protein 1
CCL3/MIP1α: chemokine (C-C motif) ligand 3/macrophage inflammatory protein 1α
CCL5/RANTES: chemokine (C-C motif) ligand 5
CD: cluster of differentiation
CXCL8/IL8: chemokine (C-X-C motif) ligand 8/interleukin 8
DMEM: Dulbecco's modified Eagle's medium
GMP: good manufacturing practice
ICAM-1: intracellular adhesion molecule-1
IL10: interleukin 10
IL4: interleukin 4
IL6: interleukin 6
MMP13: matrix metalloproteinase 13
OA: osteoarthritis
PBS: phosphate-buffered saline
PLP: platelet lysate
RGB: red/green/blue
RPS9: ribosomal protein S9
S100A8: S100 calcium binding protein A8
S100A9: S100 calcium binding protein A9
SF: synovial fibroblast
SM: synovial macrophage
TGFβ: transforming growth factor-β
TNFα: tumor necrosis factor-α
VCAM-1: vascular adhesion molecule-1
