# Supplementary figures and images for: From osteoarthritic synovium to synovial-derived cells characterization: synovial macrophages are key effector cells
Source: Arthritis Res Ther. 2016 Apr 4;18:83. doi: 10.1186/s13075-016-0983-4 (PMC4820904; doi:10.1186/s13075-016-0983-4)

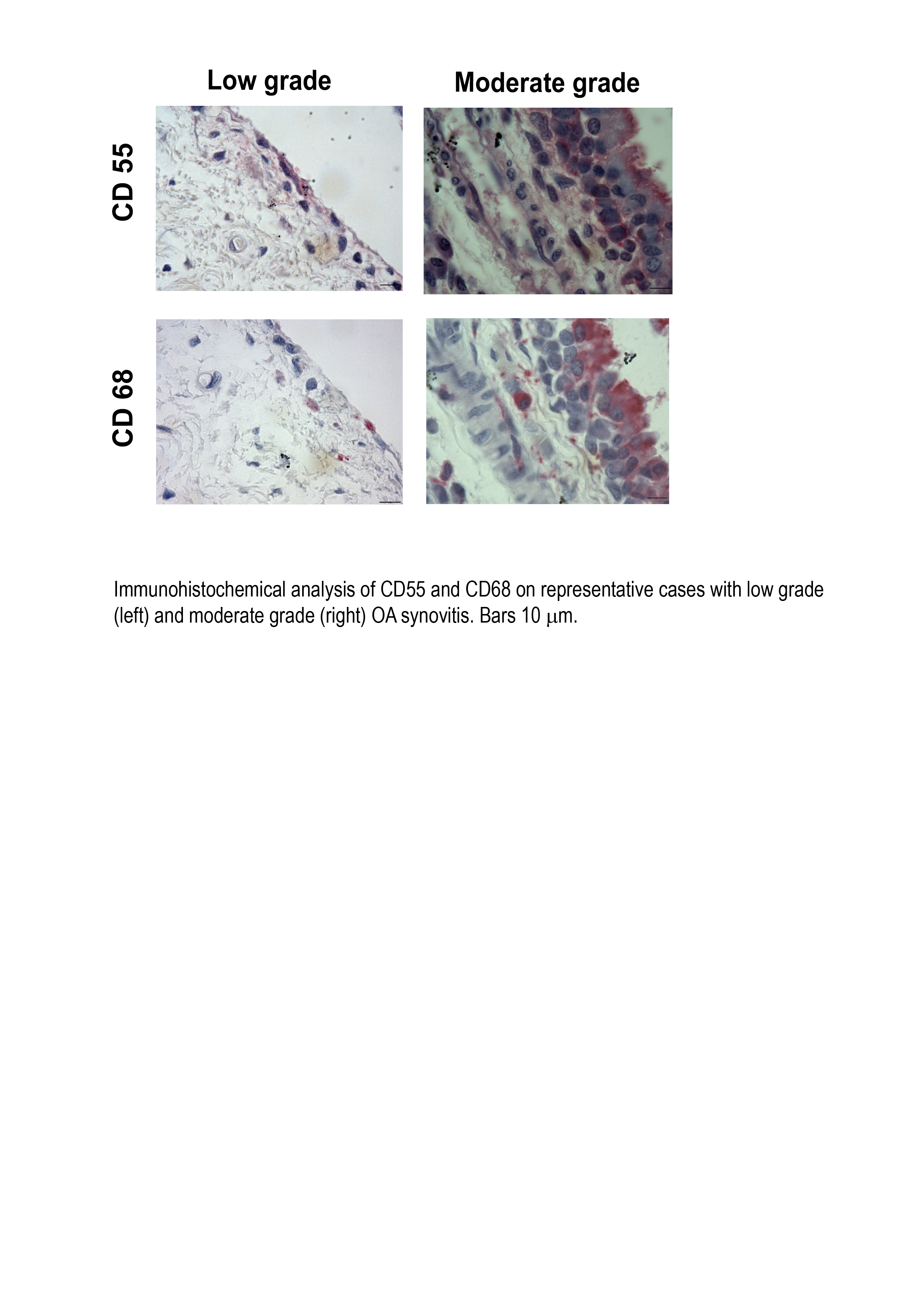

Supplement: Additional file 1: — Immunohistochemical analysis of CD55 and CD68 on representative cases with low grade (left) and moderate grade (right) OA synovitis. Bars 10 μm. (TIF 2835 kb) [file 13075_2016_983_MOESM1_ESM.tif]
